# Hygienic Legislation

**Published:** 1911-09

**Authors:** G. Henri Bogart

**Affiliations:** Terre Haute, Ind.


For Texas Medical Journal.

### Hygienic Legislation.

BY G. HENRI BOGART, M. D., TERRE HAUTE, IND.

"There are too many laws"; "Popular opinion will not allow the enforcement of laws." These are too commonly heard as the excuse of the coward, and the dolce far niente folk.

Some are satisfied to drift along in the same old groove, and they are the majority, too; looking back to the good old days of the fathers, disregarding the onward march of divinity made manifest by evolution.

It is sadly true that laws are made, and disregarded, but there was one who said, " 'Tis better to have loved, and lost, than never to have loved at all."

A law is the crystallized exponent of a need, and here, if anywhere in the economy of jurisprudence and government, do we most fully find the demonstration of the dictum of Holy Writ—"Ask and ye shall receive."

Those good people who sit supine, and then grumble that the "interests" get all the laws that they want, have but themselves to blame.

When a Legislature meets there are thousands of measures in contemplation, and the majority of the lawmakers are mere men—men with their own business most prominently in mind; men to whom the majority of the issues are new factors, and the majority of whom are honestly desirous of doing the best for their constituents.

They are the visible assets of the political party, which elected them, and the whip of partisan control cracks ever about their ears, one weakness.

That interest which sends the best attorney to this, the makers, because the issue is presented to the member in such wise that he believes it right.

Unless there is a demand for a law, especially if radical, if an innovation, the average man is apt to "pass it up."

Hygienic laws, particularly, will seem to the untrained member as a cranky matter, and will receive slight consideration unless the shibboleth of authority come with it.

Agassiz said, "The range of a human attainment is now so wide and so varied that no man may know it all, and the well-educated man of today is he who can certainly go to the correct authority, and who has the basic training to weigh the facts, when found," and the lawmaker must turn to the doctor for his information on questions of medical importance, and must accept the expert testimony as final.

For ten years Dr. Harry Sharp went to the Indiana Legislature with a band of friends, and sought to get a law to permit the sterilization of the unfit, that they might not send the burden of their heredity down to posterity, and finally on the last day of the session of 1907, and by a majority of one, the bill was passed, and from this has extended until already there are eight Commonwealths practicing this beneficent factor of racial betterment and economic release from the care of the pauper and the criminal, and the measure is growing.

The most important law of the past year is the "Utah plan," which owes its passage to Dr. Frederic Clift, editor of the Utah medical journal—a law providing for the report and quarantine of all venereal diseases.

That the venereal diseases are infectious—highly so—is not questioned by intelligent physicians, and the other fellows who through ignorance, stupidity, cupidity or general cussedness hold otherwise, are of so little importance as to be a negligible factor.

We rush up the red flag for meales, and whooping cough, self-limited, and relatively harmless, but conceal the most horrible of all—the venereals; indeed, in some of the States, the making public of the fact that one suffers from these so-called disgraceful ailments is grounds for prosecution for criminal libel.

The literal hell in connection with the venereals is the fact that the greatest sufferers are the innocent—the wife and children—contaminated through the purest, sweetest relationship of the race, the embraces of the lovelit nuptial couch.

Syphilis in particular is spread in so many indirect methods that the bearer of these germs is more dangerous to society than is the rabid dog, or the blinded viper under the glare of Sirius.

Mawkish sentimentality—prudery—hold that the presence of these specters must be ignored, and ethics would seal the lips of the medical man, even when he sees the impure bridegroom, lead pure, sweet, trusting womanhood to the altar of bestial degradation.

Remember that simple gonorrhea, the "clap, not so much to be feared as is a bad cold" (what a silly lie) may, and in truth, does become latent, and constitutional, and the victim condemns his wife to a fate far worse than death, sterility, pelvic ailments in protean form; that 80 per cent of the pelvic operation on the female come from this source.

Remember that in one year one person in five of the entire population of the great city of New York was treated for this class of disease.

Within the past year the writer has had personally to do with five cases in which the man had carried gonorrheal infection for periods ranging from fifteen years and upward, and in every case had married and the wife had been the victim.

In one case the woman had consulted as to her sterile condition, and later the husband had contracted an obstinate gonorrhea, which, I concluded, upon consultation, had been ingrafted onto a latent case, and demanded the confidence of the patient as the price of further effort to cure. He then told of a light clap a few months prior to his marriage, and protested against the thought that this had any effect upon his present condition, as he had it "dried up" in a short time. Investigation showed that the woman had been a hospital patient for obscure female trouble a few weeks after her marriage, and that her health had been impaired ever since. Incidentally, the husband had told his wife of his present dilemma, and had made her believe that he had contracted the disease "sleeping between foul sheets in a hotel in a small town," and when she asked me if such infection were not possible I replied in the affirmative, with the mental reservation of not telling her what must have been between the sheets in question at the time.

Another man came to us for sterilization with the story that his wife had suffered eleven miscarriages in the past thirteen years and that her health was all "shot to pieces," and that he was willing to submit to the operation for her sake, as he did not wish her to suffer any farther pregnancies, and as the operation for sterilization does not impair sexual powers and enjoyment he was not risking anything at that. When the scrotal sac was opened to expose the spermatic cord that the vas deferens might be severed, a general mass of adhesions was encountered, and I at once charged him with having had specific inflammation. He finally acknowledged the fact and told us of the severe orchitis that had resulted.

Bacteriological examination showed that he was still a victim of gonococci, though he was suffering no inconvenience, and was a fine looking, healthy appearing man.

But the innocent woman, broken in health, a life of invalidism, and suffering before her, what of her?

Such instances are too common to mention, still we who could prevent them and do not lift voice to prevent them are as culpable as though we had directly caused them.

We are fighting tuberculosis, yet much of the devastation of this dread disease is the indirect result of the specific infection, for the loss of vitality, the lowering of the natural resistance forms a fertile ground for the ravages of the tubercle bacilli; the black plague is the fertile hotbed for the propagation of the white plague.

Not all cases of venereal infection will be reported, not all reported cases will be quarantined, in Utah under this law, but some will, and some headway will result in the control of this terrible menace to the development of the race according to the divine plan. Then there will come a public awakening amongst the dear old vox populi as to the menace, and the law will prove educational, as well as prophylactic; educational, where knowledge is so sadly needed. The reporting and quarantining of these most loathsome of the infectious diseases is a step, and all progress in the march of development is by single steps.

There are medical men who oppose this law. Some have left the State which will seek to enforce it, but progress will not stay for individual likes, or dislikes, and the thin point of the wedge has entered.

Dr. Clift will doubtless send copies of the law and of the rules of the board of health for their enforcement to those who are sufficiently interested to forward postage for the information. His address is Salt Lake City.

He has engaged in a campaign for another radical law—one which shall compel the man seeking to obtain a marriage license to present a certificate of a bacteriological examination, showing that he is free from venereal infection before the marriage license can issue, and has entered his two journals—the doctor edits the *Denver Medical Times*, as well as the *Utah Medical Journal*—on an educational campaign for the creating of an intelligent demand for the law.

I have been requested to help in the movement, and have furnished him with five papers looking to this end.

A similar law was the only one of the eight health measures that we lost in Indiana the past winter, and it was only lost through the immensely more important (?) element of political affairs, when this session was the one next preceding a presidential election, and something had to be done to play to the galleries.

In the lower house the vote was practically unanimous, and the Senate was with us, but when the session closed by constitutional limitation the bill was about two days down the calendar.

We were almost glad that it did not pass as amended, as it was not sufficiently explicit, when the changes had been made to keep the bill from offending the tender susceptibilities of the voters.

Owing to the number of practitioners who are not competent, or who are not equipped with the necessary laboratory apparatus, we think that the examinations should be made from smears and specimens sent to a department of the State board of health, and as the marriages of Indiana average nearly eighty per day the fee need not be large to make the establishment self-sustaining.

The objection will be made that this would be another tax upon the intended bridegroom, but what does this little amount to, when measured against the monetary losses of supporting an invalid wife through all the years.

The other horrors are immeasurably greater, but one must show a dollar saving to reach the comprehension of the average lawmaker.

Oh! doctor, will you lend your influence to get laws such as these two for your community?

Of the seven laws which the State Board of Health secured one provides for the medical inspection of schools, though the bill "recommends and permits" the local trustees to act, instead of being mandatory.

Another compels the counties to set aside 5 per cent of the surplus dog tax fund—what is left after the losses of domestic animals, killed by dogs has been paid—for the establishment of a Pasteur treatment of persons exposed to rabies, and including the expenses of the mother, or nurse, in case of children.

In cases of local epidemics, or the fear of such epidemics, the local health officers may order the muzzling of all dogs, and the killing of all, in which the orders are not obeyed and all peace officers are compelled, under heavy penalty, to carry out the provision of the act.

The law as to the erection and furnishing of school buildings is very comprehensive. Good, dry walks, playgrounds, water supply, location, toilet accommodations, outbuildings, cloak rooms and heating are all provided for.

Steam heat is forbidden; the system must be hot air, with pure fresh air provided in the intake system. Should a building be erected contrary to the provisions of the law, the officer ordering it shall be fined from $100 to $500, and all bills for material and construction shall be null and void.

No building may be erected within 500 feet of any steam railroad, livery or breeding barn, noise making industry or amid unhealthful conditions.

Annual cleaning and disinfection is particularly described. Janitors and teachers who are syphilitic, tubercular or intemperate, or addicted to drugs, may not be employed, and if so employed the contract is void.

This bill provides for the full teaching of the principle of sanitary science, and infection, from leaflets to be prepared jointly by the boards of health and education.

The Ophthalmia Neonatorum bill compels the treatment of the eyes of the newly-born, with a report to the local health officer, and in addition to the fine for non-compliance voids the bills for professional service in the case affected.

On cold storage, it is required that all goods that have been in cold storage shall be marked with the date of storage, which marking shall be plainly displayed with the goods until its ultimate disposition; that no goods shall be sold after nine months of cold storage, failing which it shall be destroyed; license are required; all cold storage plants are under the supervision of the boards of health, and the bill requires local health commissioners to examine a specifically described record, at intervals; and constitutes each day of violation as a separate offense, as well as holding responsible all employes as fully responsible as owners.

The State Food and Drug Commissioner is made the State Commissioner of Weights and Measures, with all local health officials ex-officio deputies, and provision is made for sealers for counties and cities.

Cities of the first class are compelled to equip and to maintain public baths, comfort stations, and playgrounds; all school property shall be so used during vacation, and the right of eminent domain is extended to apply to the securing of adequate grounds for such purposes.

We consider that with the Legislature juggling the liquor question as a political asset, we were very fortunate in Indiana, though much of the good accomplished arose from the wise skill and direction of Dr. J. N. Hurty.

When Dr. Hurty, as Secretary of the State Board of Health, first took charge of the office he became probably the most hated man in the State, for his cranky interference, as it was called, but fortunately his position was such that politics could not oust him, and today he is as potent a power for good as is any man in the State, and he keeps everlastingly hammering at unsanitary conditions, and, having educated the people, they have come to love, honor and believe him.

Both Texas and Oklahoma fought for the Indiana plan for the sterilization of the unfit, the epileptic, the incurably insane, the habitual criminal, and the like, and though the bills failed in both States, for lack of time to stop playing politics, the educational propaganda assures the passage in the next session.

Such are some of the laws for the betterment of mankind that fell from the grist of the past winter.

Every one of these laws needed some man single of purpose to enable it to become a fact; each measure needed doctors in the local communities to build the popular demand for these laws.

Dear reader, join us; write for copies of the measures which appeal to you, and find rest and mental growth in the exercise.

---

For Texas Medical Journal.

